# Spatio-temporal regulation of EGFR signaling by the Eps15 homology domain-containing protein 3 (EHD3)

**DOI:** 10.18632/oncotarget.13008

**Published:** 2016-11-01

**Authors:** Mohamed Amessou, Abdul Shukkur Ebrahim, Ashok Dilly, Melvin Joseph, Marina Tabolina, Sahiti Chukkapalli, Louay Meroueh, Joseph T. Syed, Allison Liddane, Siera Lanae Lang, Ayad Al-Katib, Mustapha Kandouz

**Affiliations:** ^1^ Department of Pathology, Wayne State University School of Medicine, Detroit, Michigan, USA; ^2^ Lymphoma Research Lab, Wayne State University School of Medicine, Detroit, Michigan, USA; ^3^ Karmanos Cancer Institute, Wayne State University, Detroit, Michigan, USA

**Keywords:** EHD3, EGFR, glioblastoma, signaling

## Abstract

The epidermal growth factor (EGF) receptor EGFR is a major receptor tyrosine kinase whose role in gliomagenesis is well established. We have recently identified EHD3 [Eps15 homology (EH) domain-containing protein 3], an endocytic trafficking regulatory protein, as a putative brain tumor suppressor. Here, we investigate the underlying mechanisms, by establishing a novel mechanistic and functional connection between EHD3 and the EGFR signaling pathway. We show that, in response to stimulation with the EGF ligand, EHD3 accelerates the rate of EGFR degradation by dramatically increasing its ubiquitination. As part of this process, EHD3 also regulates EGFR endosomal trafficking by diverting it away from the recycling route into the degradative pathway. Moreover, we found that upon EGF activation, rather than affecting the total MAPK and AKT downstream signaling, EHD3 decreases endosome-based signaling of these two pathways, thus suggesting the contribution of EHD3 in the spatial regulation of EGFR signaling. This function explains the higher sensitivity of EHD3-expressing cells to the growth-inhibitory effects of EGF. In summary, this is the first report supporting a mechanism of EHD3-mediated tumor suppression that involves the attenuation of endosomal signaling of the EGFR oncogene.

## INTRODUCTION

The National Cancer Institute (http://www.cancer.gov/cancertopics/types/brain) reports an estimated 22,850 new cases and 15,320 deaths from brain and other Central Nervous System (CNS) cancers in the US in 2015. Worldwide, in 2012, there were 256,000 new cases diagnosed (http://www.wcrf.org/int/cancer-facts-figures/worldwide-data). Among primary intracranial tumors, gliomas are the most frequent. The World Health Organization (WHO) classification distinguishes between low (I and II) and high (III and IV) grade gliomas [1]. Grade IV astrocytomas, also known as glioblastomas or glioblastoma multiforme (GBM) constitute the highest and most prevalent grade [2]. GBM is a poorly differentiated astrocytic tumor, highly heterogeneous, extremely invasive and showing a complex biology [3]. GBM is characterized by an amplification and/or mutation of wild-type epidermal growth factor receptor (*EGFR*), amplification of the platelet-derived growth factor (*PDGF*) and receptors (*PDGFRα/β*), mutations of the *IDH1* and *IDH2* genes or loss of tumor suppressor genes such as *p53*, *PTEN* or *p16^Ink4a^*. *EGFR* aberrations are the most widespread oncogenic events in GBMs, with a frequency of over 50% [4]. EGFR is known as a key Receptor Tyrosine Kinase (RTK) and a therapeutic target in many cancers including gliomas [5–7].

We have recently identified *Ehd3* as a new putative glioma tumor suppressor, whose loss of expression is a very frequent event in gliomas of all grades [8]. The EHD3 protein belongs to the group of C-terminal Eps15 homology domain-containing (EHD) proteins, a relatively newly identified highly conserved family of proteins involved in endocytic trafficking. The EH domain is a motif of ~100 residues, typically found at the N-terminus of many proteins. However, in mammals, the EHD family of proteins has the EH domain at the C-terminus. This family of four paralogs (EHD1-EHD4) has been implicated in receptor intracellular trafficking, namely in internalization and recycling to the plasma membrane [9, 10]. In particular, although information is scarce, EHD3 was shown to be involved in early-endosome-to-recycling-endosome transport [11] and in the regulation of endosome-to-Golgi transport [12].

In this study, we sought to determine whether EHD3 regulates the trafficking, signaling and function of EGFR. It is well acknowledged that endocytosis and vesicular trafficking have an important role in regulation and integration of RTK signaling pathways and functions [13–18]. Therefore, it is not surprising that these essential biological processes are involved in cancer progression [19–21]. In particular, much effort is dedicated to identifying the mechanisms and proteins involved in EGFR trafficking in signal modulation, which remain largely unknown [17, 22]. Here we describe data showing that EHD3 regulates EGFR expression, activation, signaling and signal attenuation upon ligand stimulation. We show that by accelerating EGFR ubiquitination and sorting from the endosomes into a lysosomal degradation compartment, EHD3 has a specific inhibitory effect on Akt and ERK endosomal signaling, which could contribute to growth-inhibitory effects of high dose EGF ligand stimulation.

## RESULTS

### EHD3 expression increases EGFR base levels in the absence of ligand stimulation

**Figure 1:**
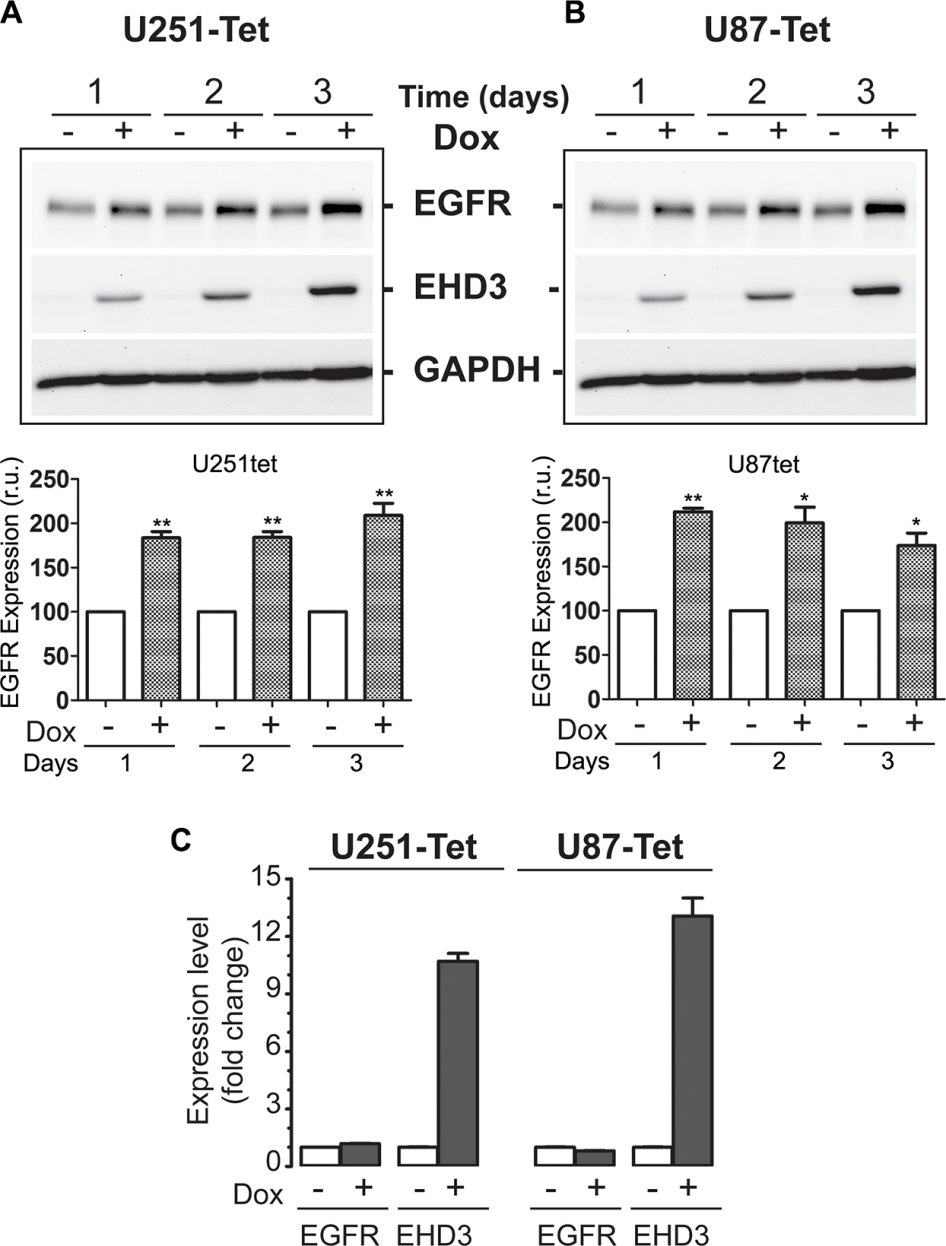
In absence of ligand stimulation, EHD3 increases the level of EGFR in glioblastoma cells (**A**) Protein extracts from U251tetEhd3 cells induced (+) or not (−) with Dox, for 1– 3 days, were examined by immunoblotting for EGFR expression. (**B**) The same analysis was also performed on U87tetEhd3 cells. Histograms depict densitometric quantitation of 3 different blots each and the results are shown as relative units (r.u.) in comparison to the control without Dox being the 100% value. (**C**) Real-time RT-PCR analysis of control (−) or Dox-induced (+) U251tetEhd3 and U87tetEhd3 cells shows that EHD3 expression does not affect EGFR expression at the transcript level. Results are shown as fold change in expression in Dox-induced *versus* the control without Dox treatment.

We have recently shown evidence that EHD3 possesses tumor suppressor functions in gliomas [8]. In light of the role of the EHD family of proteins in endocytic trafficking [9, 23], we hypothesized that at least parts of EHD3′s functions might be mediated by regulating the trafficking of receptor tyrosine kinases (RTKs), and thus their signaling ability and functions. EGFR is known as a key RTK and leading therapeutic target in many cancers including gliomas [5–7]. We thus elected to assess whether EHD3 regulates the fate of EGFR. Using a Dox-inducible system, we examined the impact of restoring EHD3 expression to two glioma cell lines that express very low levels of EHD3, *i.e.* the U251 and U87MG cells, on the expression of EGFR. Contrary to our expectation, the expression of EHD3 in U251 cells resulted in higher levels of the EGFR protein, as early as 1 day after Dox induction, with the effect persisting at least 3 days later (Figure 1A). This effect was also observed in U87MG cells (Figure 1B). When examining the EGFR mRNA transcript by real time RT-PCR, we found no significant differences in EGFR expression between Dox-induced and –non induced control cells (Figure 1C), suggesting that the effect of EHD3 on EGFR expression is post-transcriptional and involves an increase in the EGFR protein levels.

### EHD3 decreases EGFR ubiquitination in the absence of ligand stimulation

**Figure 2:**
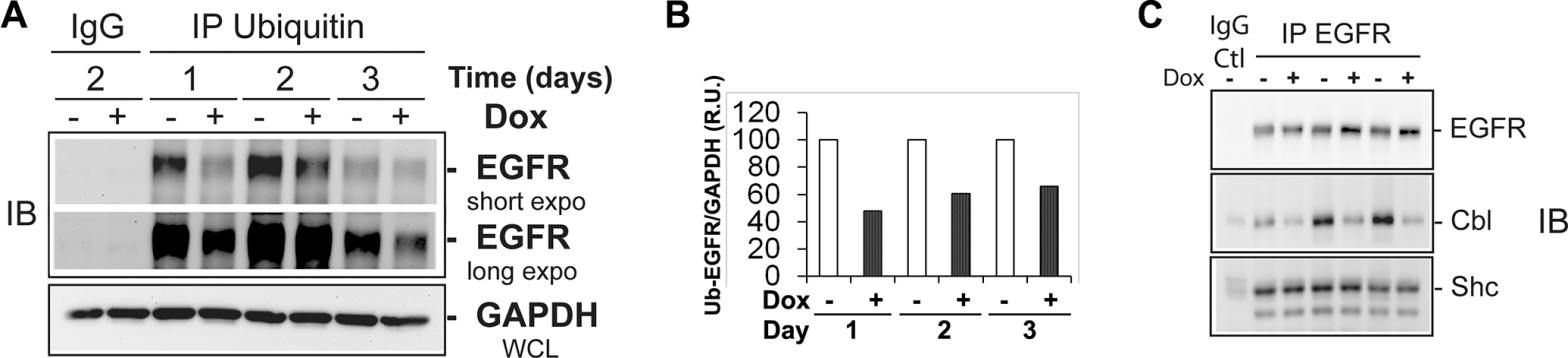
In absence of ligand stimulation, increased levels of EGFR in EHD3-expressing cells are associated with decreased ubiquitination and less recruitment of the protein Cbl (**A**) Protein extracts from U251tetEhd3 cells induced (+) or not (−) with Dox for 1–3 days, were used to immunoprecipitate (IP) ubiquitinated proteins and subsequently assessed by immunoblotting (IB) using an anti-EGFR antibody. A longer exposure is shown for a better appreciation of the effects at day 3. The IgG IP control was performed with samples collected on day 2. (**B)** The histogram depicts densitometric quantitation of a representative immunoblot and the results are shown as relative units (r.u.) of the EGFR/GAPDH ratio in Dox-induced *versus* the control without Dox treatment, the latter being at a 100% value. **C)** An EGFR IP was performed on similar samples as in A, and subject to immunoblotting to assess levels of EGFR, Cbl and Shc.

Protein accumulation could be regulated by multiple post-transcriptional mechanisms, among which post-translational modification by ubiquitination is among the most prevalent. We hypothesized that EHD3 might prevent EGFR degradation by increasing the level of its ubiquitination. We thus examined the effect of EHD3 expression on the ubiquitination of EGFR. Immunoprecipitation of total ubiquitinated proteins followed by immunoblotting with an anti-EGFR antibody revealed that increases in EGFR levels are associated with lower levels of ubiquitination (Figure 2A, 2B), thus suggesting that in absence of EGFR ligand stimulation, EHD3 stabilizes EGFR by decreasing its ubiquitination. Furthermore, we analyzed the binding of EGFR to Cbl, a known regulator of this receptor's ubiquitination [22, 24]. Using co-immunoprecipitation, we found that EHD3 expression dramatically decreases the levels of Cbl constitutively associated with EGFR, while the levels of Shc adaptor association with EGFR are not affected (Figure 2C). Therefore, our data suggest that EHD3 increases the base level of EGFR, by affecting the level of constitutively recruited ubiquitination machinery proteins.

### EHD3 attenuates ligand-dependent activation of EGFR

**Figure 3:**
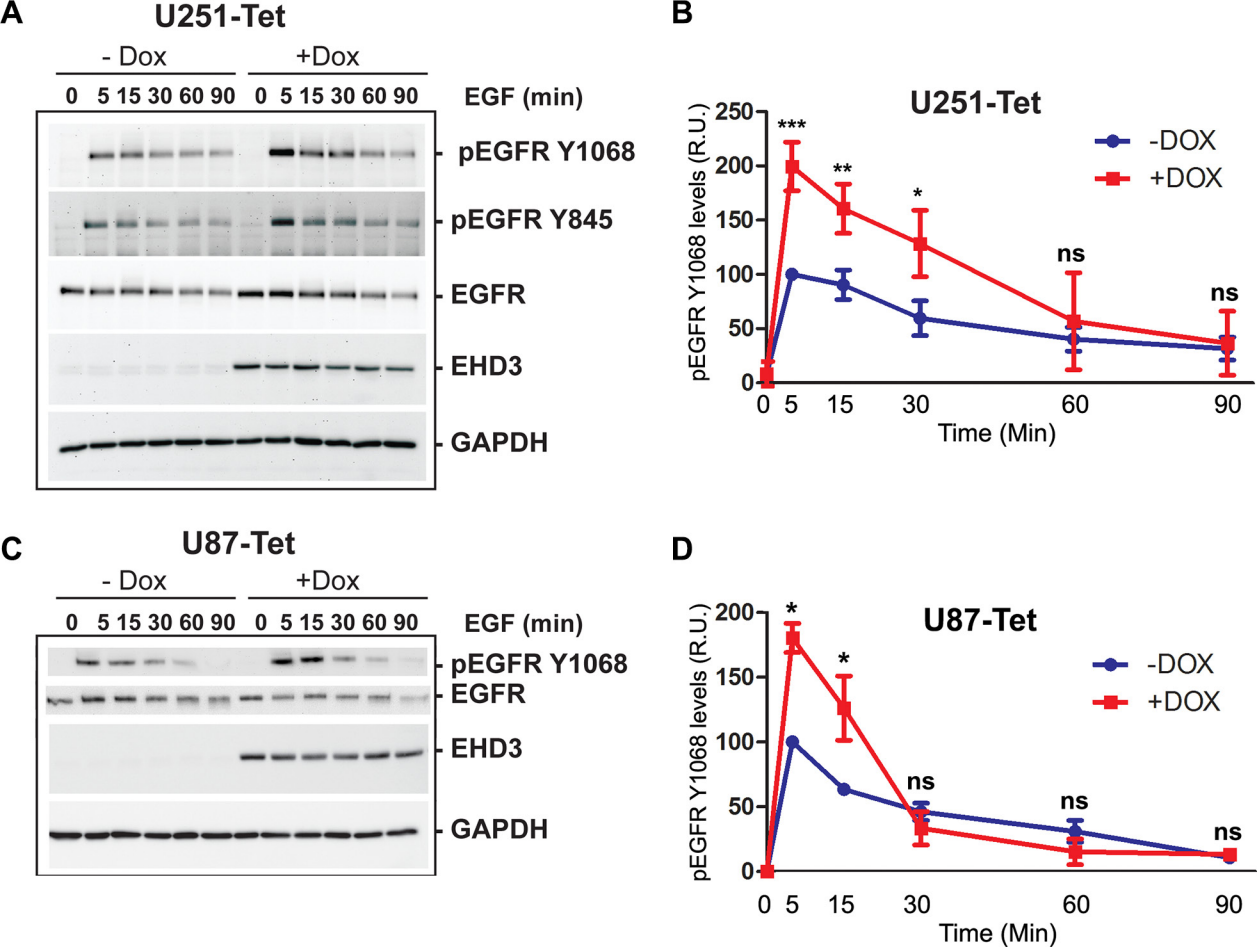
Upon EGF ligand stimulation, EGFR signal attenuation is more pronounced in EHD3-expressing cells (**A**) U251tetEHD3 cells were induced (+Dox) or not (−Dox) to express EHD3 by Dox treatment, serum starvation-primed overnight prior to stimulation with EGF at different time points. Whole cell lysates were analyzed by immunoblotting for the levels of total EGFR, tyrosine phosphorylated EGFR (pEGFR), EHD3 and GAPDH. (**B**) Densitometric quantitation of the signal intensities were quantified using the ImageJ software (NIH) from three different immunoblots and presented as relative units (r.u.). (**C**) The kinetic of EGFR activation and signal attenuation was similar in U87tetEhd3 cells. (**D**) Similarly, the kinetic of EGFR phosphorylation was analyzed by densitometry measurements and presented as relative units (r.u.). Statistical significance was calculated with a two-tailed *t* test using GraphPad Prizm 5.0. ns: not significant, *p < 0.05, **p < 0.01, ***p < 0.001.

We next tested the hypothesis that the function of EHD3 might be different in presence of EGFR ligand activation, by assessing the impact of EHD3 expression on EGF-mediated activation of EGFR. As described above, in absence of EGF stimulation (Time 0), EHD3 increases the level of total EGFR (Figure 3A). Upon treatment with EGF (20 ng/ml), this higher EGFR expression level immediately translates into a burst of higher EGFR phosphorylation levels at either tyrosine Y845 or tyrosine Y1068 after 5 minutes (Figure 3A, 3B). However, this initial peak is followed by a decrease in EGFR phosphorylation that is more dramatic in EHD3-expressing cells than their control counterparts. This is evidenced by the linear regression analysis of the attenuation of EGFR phosphorylation following the 5 min peak (Supplementary Figure S1A). In parallel, the total level of EGFR is decreased more rapidly when EHD3 expression is restored than in the EHD3-devoid control (Supplementary Figure 1B). These effects were also observed in U87MG cells (Figure 3C, 3D). In other respect, although EHD3 appears to also increase EGFR activation by low dose EGF, the effects are less strong (Supplementary Figure S2).

**Figure 4:**
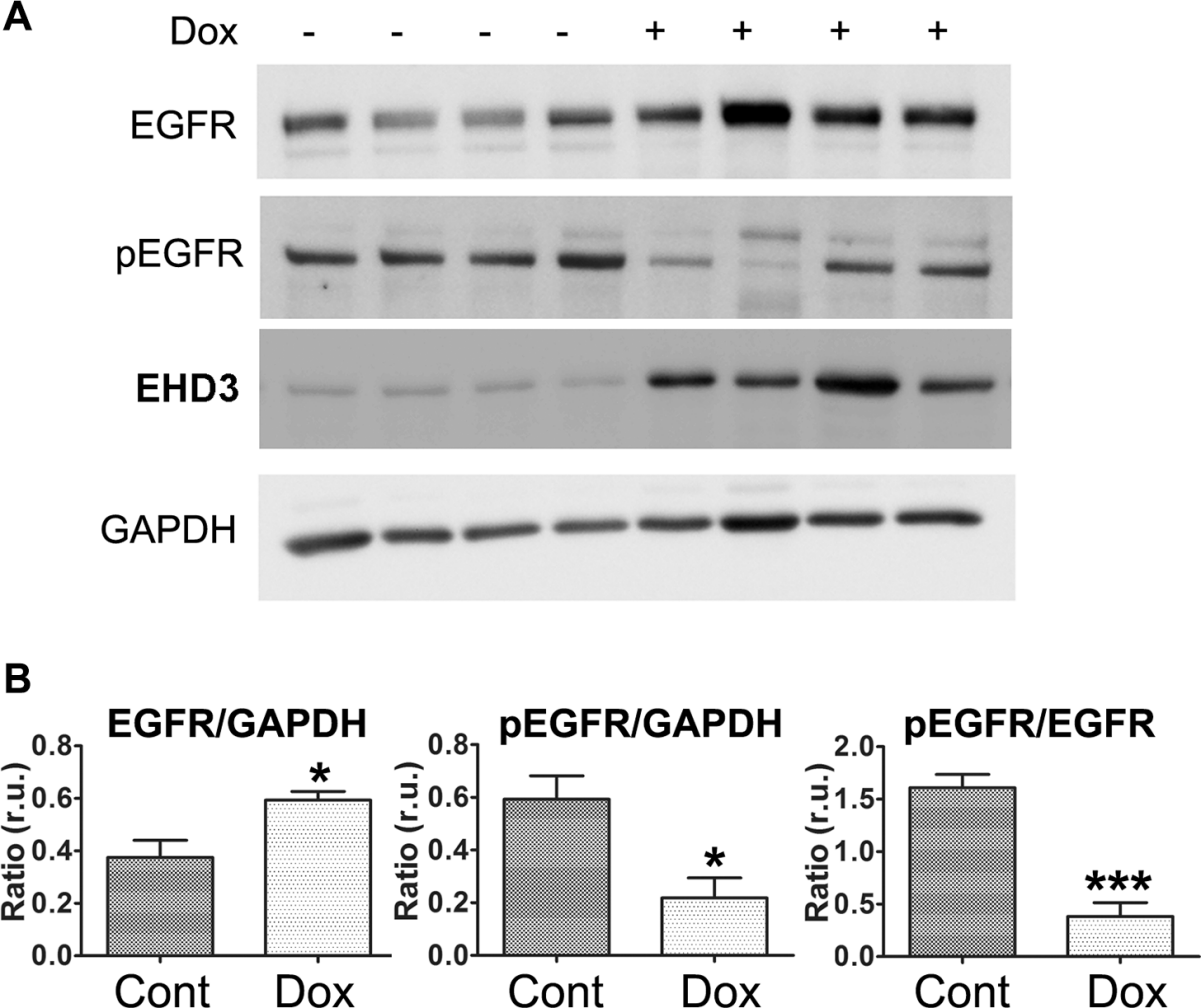
EHD3 expression inactivates EGFR *in vivo* (**A)** U251tetEhd3 cells were grown as xenografts injected sub-cutaneously to nude mice given (+) or not (−) Dox in drinking water at 2 mg/ml [8]. Tumors were retrieved from 4 mice each group and whole cell lysates were analyzed by immunoblotting for the levels of total EGFR, tyrosine phosphorylated EGFR (pEGFR), EHD3 and GAPDH. (**B**) Signal intensities were quantified using the Image Studio Lite software (LI-COR Biosciences) and the indicated ratios presented as relative units (r.u.). Statistical significance was calculated with a two-tailed *t* test using GraphPad Prizm 5.0. *p ≤ 0.05; ***p ≤ 0.001.

We have previously reported, using a xenograft model of U251tetEHD3 cells implanted subcutaneously in nude mice, that EHD3 expression reduces tumor growth *in vivo* [8]. To examine whether EHD3 affects EGFR levels and activation *in vivo*, we extracted proteins from xenograft tumors upon retrieval from the animals and we found that while Dox-induced expression of EHD3 increased the levels of EGFR, it significantly decreased the fraction of phosphorylated EGFR (Figure 4). These data suggest that when cells are allowed to form tumors *in vivo*, with access to activation by the animal's growth factors, EHD3 reduces EGFR phosphorylation. Therefore, this finding provides an *in vivo* validation of the results we have obtained *in vitro* and allows us to conclude that EHD3 attenuates ligand-dependent EGFR activation.

### EHD3 decreases constitutive activation of mutant EGFRvIII

We next asked whether EHD3 would affect the activation of a ligand-independent form of EGFR. The EGFRvIII mutant is the result of a deletion of exons 2–7 of the EGFR gene that produces a protein with a 267 amino acids deletion in the ligand-binding domain, rendering the receptor unable to bind the ligand. Subsequently, activation of EGFRvIII is low but constitutive [25]. Using a DOX-inducible system, we expressed EGFRvIII in U251MG cells and concomitantly transfected with a control plasmid or a plasmid encoding EHD3. The EGFRvIII receptor (molecular weight 145 kDa) is constitutively phosphorylated in control pcDNA-transfected cells (Supplementary Figure S4). However, when cells were transfected to express EHD3, we observed a strong decrease in EGFRvIII constitutive phosphorylation (Supplementary Figure S3). This result suggests that EHD3 decreases constitutive activation of EGFRvIII.

### EHD3 regulates the EGFR endosomal signaling and sensitizes glioma cells to EGF-induced growth inhibition

**Figure 5:**
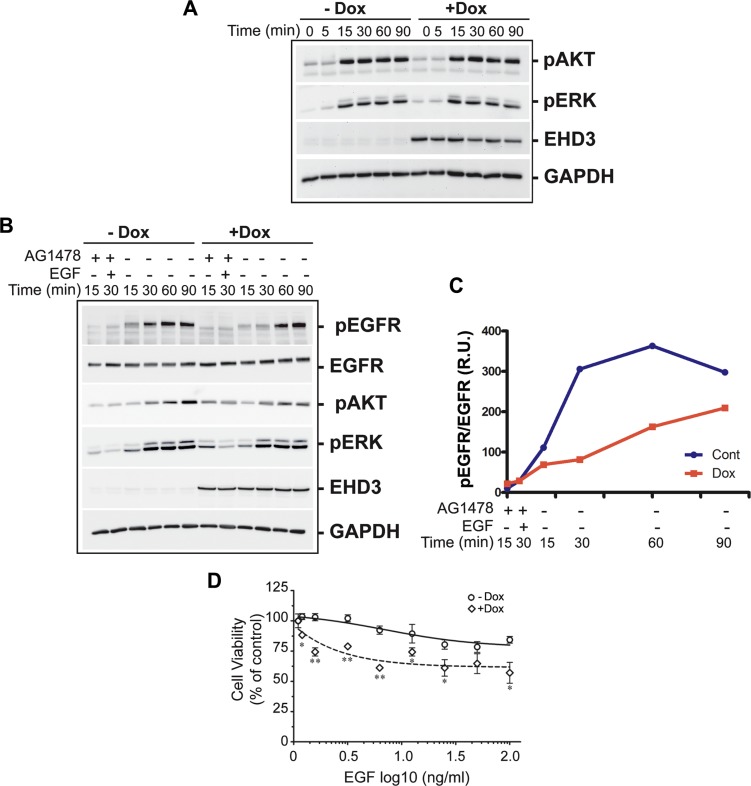
EHD3 modulates endosomal signaling and growth inhibitory effects of EGF ligand stimulation (**A**) EHD3 minimally affects total AKT and ERK signaling pathways upon ligand stimulation. U251tetEHD3 cells were induced (+Dox) or not (−Dox) to express EHD3 by Dox treatment, serum starvation-primed overnight prior to stimulation with EGF at different time points. Whole cell lysates were analyzed by immunoblotting for the levels of phosphorylated AKT (pAKT), phosphorylated ERK1/2 (pERK), EHD3 and GAPDH. (**B**) Selective activation of endosomal EGFR results in significant inhibition of AKT and ERK signaling. U251tetEhd3 cells were serum starved for 24 h, pre-treated with the EGFR tyrosine kinase inhibitor AG1478, prior to treatment on ice with EGF, thus allowing the endosomal internalization of non-activated EGF-EGFR complexes without triggering membrane-originated signaling. The endosomal EGFR signaling was subsequently activated by removing AG-1478 from the medium and incubating at 37C. Whole cell lysates from different time points were analyzed by immunoblotting for the levels of total EGFR, phosphorylated EGFR, pAKT, pERK, EHD3 and GAPDH. (**C**) Densitometric quantitation of a representative immunoblot. The results are shown as relative units (r.u.) of the pEGFR/EGFR ratio in Dox-induced *versus* the control without Dox treatment. (**D**) EHD3 sensitizes glioma cells to EGF-induced growth inhibition. U251tetEhd3 cells induced (+Dox) or not (−Dox) to express EHD3, were treated with an increasing dose of EGF for 48 h and an MTT cell growth assay was performed. The mean values and standard deviation are plotted as percentages of the levels in absence of EGF treatment. The assay was performed in triplicates and statistical significance was calculated with a two-tailed *t* test using GraphPad Prizm 5.0. *≤ 0.05; **≤ 0.01.

We next asked if the effect of EHD3 on attenuating EGFR phosphorylation is reflected in downstream canonical EGFR effector signaling pathways, *i.e.* ERK and AKT. Cell extracts from U251tetEhd3 cells were analyzed after serum starvation and stimulation with EGF (20 ng/ml). Surprisingly, following ligand stimulation, we observed that the phosphorylation of both AKT and ERK was only marginally impacted by EHD3 expression (Figure 5A). This contrasts with the much stronger effect of EHD3 observed for EGFR activation.

Based on the function of EHD3 in intracellular trafficking, we thus questioned whether EHD3 affects any of these signaling pathways in a spatially-regulated manner. Indeed, differential signaling from various subcellular compartments is an important, although not very well studied, process. In particular, it has been shown that EGFR can undergo endosomal signaling [13, 14, 17, 26]. To test our hypothesis, we adapted our protocol from an approach previously described by Wang *et al*, to selectively inhibit the signaling that originates from the cytoplasmic membrane, to allow specific examination of the endosomal signaling of EGFR [27]. To this end, cells were serum starved for 24 h, pre-treated for 15 min with 0.5 μM of AG1478, an EGFR tyrosine kinase inhibitor, treated on ice with EGF (20 ng/ml). This allows the internalization of non-activated EGF-EGFR complexes into endosomes, without triggering membrane-originated signaling. The endosomal EGFR signaling was subsequently activated by removing AG-1478 from the medium (Supplementary Figure S4). As a result, we found that the endosomal activation of EGFR, assessed by its ligand-dependent phosphorylation is significantly diminished and the peak of activation delayed by at least 30 min when EHD3 is expressed (Figure 5B, 5C). As a consequence, activation of ERK and even more so of AKT was significantly decreased in presence of EHD3 (Figure 5B). Taken together, these data show that EHD3 attenuates EGFR endosomal signaling rather than the total signaling, thus suggesting a spatial regulation of EGFR signaling by EHD3.

It is particularly challenging to develop tools to specifically address compartmentalized signaling and its functional significance. Interestingly however, endosomal accumulation of the activated EGFR has been associated with growth inhibitory effects. In fact, although EGF is known for its mitogenic functions, it has early been reported that in many cell lines and contexts it can also induce cell growth inhibition [28–32]. Therefore, to examine the functional impact of EHD3-regulated EGFR endosomal signaling, we performed an MTT cell growth assay, following treatment with increasing concentrations of the EGF ligand. We observed that U251tetEHD3 cells that are not induced to express EHD3 are sensitive to growth inhibitory effects of high concentrations of EGF (Figure 5D). Importantly, this effect was substantially exacerbated when EHD3 was expressed (Figure 5D). This data suggests that EHD3 regulation of EGFR endosomal signaling modulates the cell growth response to EGF stimulation.

### EHD3 regulates EGFR trafficking upon ligand stimulation

**Figure 6:**
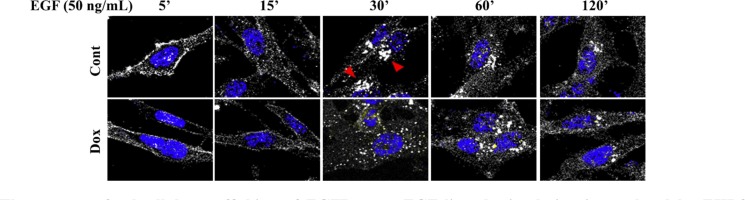
The pattern of subcellular trafficking of EGFR upon EGF ligand stimulation is regulated by EHD3 U251tetEhd3 cells were plated on glass coverslips in presence (Dox) or not (Cont) of Dox. They were incubated with an anti-EGFR antibody (Genentech) prior to stimulation with EGF for the indicated time points. Cells were then fixed, permeabilized, and stained with an alexafluor-594-conjugated secondary antibody, along with DAPI for nucleus visualization. The cells were visualized with a Leica TCS SP5 Laser (Point) Scanning Confocal microscope. Red arrowheads in control samples at 30 min indicate EGFR accumulation in the perinuclear recycling compartment, which is quasi-absent from the Dox-induced counterpart.

Subsequently, we examined whether EHD3 affects the endocytic trafficking of EGFR. To this end, we performed an antibody uptake experiment where cells were incubated with the anti-EGFR antibody prior to addition of EGF at 20 ng/ml. Upon EGF ligand stimulation, we observed that the pattern of EGFR trafficking is altered by EHD3 expression in U251 cells (Figure 6). The pattern was sensibly similar in control and Dox-induced cells 5 and 15 min after EGF stimulation, with a typical membranous staining and endosomal internalization respectively. However, while in the control cells, dense perinuclear EGFR accumulation in the recycling compartment is observed at 30 min before sorting to more individualized vesicles at 60 min, the pattern of trafficking appears different when EHD3 is expressed, with the absence of perinuclear accumulation and the appearance of individualized vesicles as early as 30 min (Figure 6). These data suggest that EHD3 attenuates EGFR signaling by either limiting its sorting towards or accelerating its exit from the endocytic recycling compartment (ERC).

**Figure 7:**
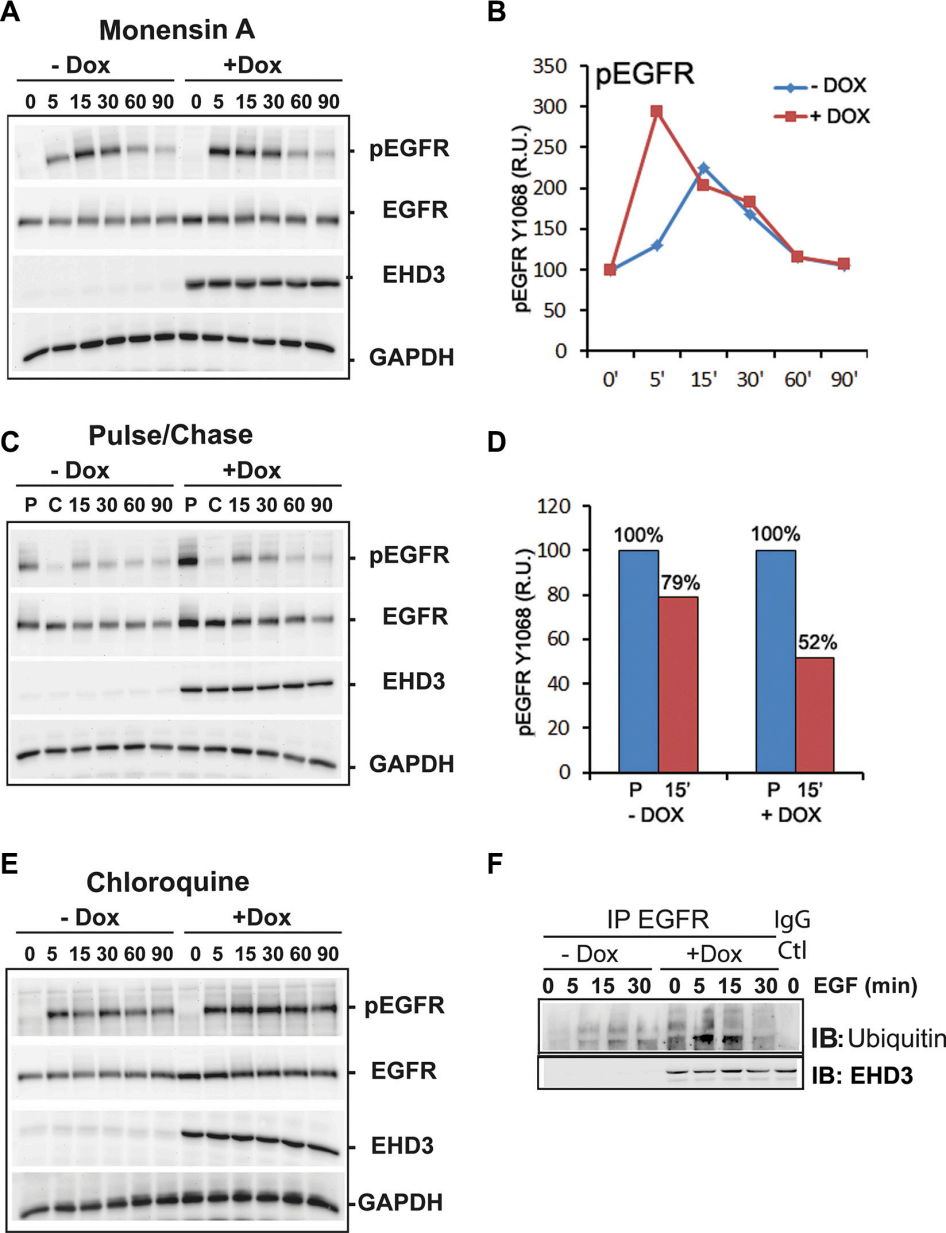
EHD3 increases ligand-induced EGFR ubiquitination and lysosomal degradation (**A**) Blockade of recycling events shifts the peak of EGFR activation only in absence of EHD3. U251tetEHD3 cells, induced (+Dox) or not (−Dox) to express EHD3 by Dox treatment, and serum starvation-primed overnight, were pre-treated with Mon A and CHX for 30 min prior to performing an EGF stimulation kinetic. (**B**) Densitometric quantitation of a representative immunoblot. The results are shown as relative units (r.u.) of the pEGFR levels in Dox-induced *versus* the control without Dox treatment. (**C**) Pulse-chase analysis of EGFR recycling. U251tetEHD3 cells were induced (+Dox) or not (−Dox) to express EHD3 by Dox treatment, serum starvation-primed overnight. Cells were subsequently stimulated with EGF and pulsed for 15 min, followed by a 60 min chase, before a second EGF stimulation for the indicated times. (**D**) The histogram depicts the densitometric quantitation of a representative immunoblot. The results are shown as relative units (r.u.) of the pEGFR levels in Dox-induced *versus* the control without Dox treatment, after either the pulse or the 15 min ligand stimulation. (**E**) Blockade of lysosomal degradation significantly blocks attenuation of EGFR signal. Similar to C, the lysosome inhibitor Chloroquine was used in presence of CHX, to pre-treat U251tetEhd3 cells prior to EGF stimulation. In A–C, whole cell lysates were analyzed by immunoblotting for the levels of total EGFR, tyrosine phosphorylated EGFR (pEGFR), EHD3 and GAPDH. (**F**) EHD3 promotes ligand-dependent ubiquitination of EGFR. Dox-induced and control U251tetEhd3 cells were stimulated with EGF for different time points and whole cell extracts were used to immunoprecipitate EGFR and immunoblot with an anti-Ubiquitin antibody.

The functions of EHD3 in subcellular trafficking are still poorly understood. Based on the reported role of EHD proteins in endosomal recycling, we sought to elucidate the trafficking events involved in the effects of EHD3 on the attenuation of EGFR activation, using pharmacological inhibition. To examine the role of trafficking in EHD3-driven attenuation of EGFR activation, we used Monensin A (Mon A), a compound known to inhibit vesicular trafficking [33, 34]. Cells were pre-treated with Mon A (100 μM), in presence of CHX (10 μM), for 30 min prior to performing an EGF stimulation kinetic. Mon A is known to block the endocytosed receptors from exiting the endocytic recycling compartments (ERC) towards either recycling or degradation, thus causing their accumulation in the perinuclear-localized ERC. Subsequently, we found that EGFR activation remains higher in Dox-induced cells than their control counterparts at 5 min stimulation (Figure 7A), similar to what we described above (*c.f.* Figure 3). However, in later time points, Mon A pre-treatment of control cells increased and prolonged EGFR phosphorylation causing a shift in the maximum activation peak from the regular 5 min to 15 min (Figure 7A, 7B). This is expected since Mon A treatment results in a longer residence of the phosphorylated receptors in endosomal compartments and delays signal attenuation. Nevertheless, in EHD3-expressing cells, this effect of Mon A is not observed (Figure 7A, 7B) and the time kinetic of signal attenuation is restored to normal (similar to what is observed in Figure 3A). This data suggests that the effect of Mon A in delaying exit from the ERC is counteracted by EHD3. By so doing, EHD3 seems to enhance the process of attenuation of EGFR activation. This could be explained by a parallel effect of EHD3 on the rate of sorting of endosomal EGFR towards the degradation compartment, which would thus cancel any endosomal accumulation. In support of this explanation is the observation that, upon ligand stimulation, the rate of decrease in levels of total EGFR is higher in Dox-induced cells (Supplementary Figure S1B). In other words, the shift in the peak of EGFR phosphorylation from 5 to 15 min is not observed in Dox-induced cells because EHD3 induces EGFR degradation so fast that it does not accumulate in endosomes, let alone get recycled. In conclusion, Mon A unravels a strong role for EHD3 in EGF-dependent EGFR degradation.

To further support these data suggesting a role in degradation rather than recycling of EGFR, we asked whether, following ligand activation and internalization, the fraction of EGFR that is recycled back to the membrane would be affected by EHD3. To this end, we performed a pulse/chase experiment whereby cells are subject to a 15 min pulse, followed by a 60 min chase, before treating again with EGF for various times. The ligand stimulation experiments were performed in presence of cycloheximide (CHX, 10 μM) to block protein neo-synthesis. In Dox-induced cells, the strong initial EGFR phosphorylation peak observed after the pulse (P) is not subsequently restored even after a 60 min chase (C) and 15 min ligand stimulation, with a proportion of 79% activation in control *versus* 52% in EHD3-expressing cells (Figure 7C, 7D). The results suggest that in presence of EHD3, as a result of the first EGF pulse, most of the ligand-activated EGFR is not recycled back to the membrane and is thus no longer available for a second round of activation (Figure 7C, 7D). A direct effect on recycling would have caused the accumulation of phosphorylated EGFR in endosomes, which would have been reflected as a higher level of activated EGFR after the chase. This is contrary to the observed near disappearance of the phosphorylated EGFR signal (Figure 7A). The data thus suggests that in this pulse-chase experiment, upon EGF stimulation, EHD3 causes the degradation of EGFR rather than preventing it from recycling.

### EHD3 increases ligand-induced ubiquitination and lysosomal degradation of EGFR

To further analyze the role of EHD3 in ligand-dependent degradation of EGFR, we used the lysosome inhibitor Chloroquine. Pre-treatment with Chloroquine (25 μM, 30 min) in presence of CHX (10 μM), resulted in inhibition of the ligand-induced EGFR degradation and significantly blocked EGFR inactivation in both Dox-treated and untreated cells (Figure 7E), thus confirming that EHD3 plays a major role in the attenuation of EGFR activation via enhancing the lysosomal degradative process. Since ubiquitination has been shown to be a critical post-translational modification for EGFR internalization, endosomal sorting and degradation [24], we analyzed whether the effect of EHD3 on ligand-induced EGFR degradation could involve an effect on ubiquitination. As shown in Figure 7F, Dox-induced and control U251tetEhd3 cells were stimulated with EGF (20 ng/ml) for different time points and protein extracts were used to immunoprecipitate EGFR and immunoblot with an anti-Ubiquitin antibody. We observed a drastic increase in EGFR ubiquitination, 5 min following EGF activation in Dox-induced U251tetEhd3 cells, which subsequently drops to base levels after 30 min. This effect was much less prominent in the control counterparts (Figure 7F). This data suggests that EHD3 promotes lysosomal degradation of EGFR by increasing ligand-dependent ubiquitination.

## DISCUSSION

In a recent work, we have shown that *Ehd3* presents features of a glioma tumor suppressor gene [8]. As a follow up mechanistic investigation, and based on the reported role of the EHD family of proteins in endocytic trafficking [9, 23], we hypothesized that EHD3 might undergo its functions via regulating the trafficking of receptor tyrosine kinases (RTKs), and thus their signaling ability and functions. Since EGFR is a key RTK involved in gliomagenesis [5–7], it was a logical step to ask whether EHD3 is involved in defining the fate of EGFR signaling. The first hint at the complexity of the answer to this question was our unexpected finding that the expression of EHD3 in either U251 or U87MG cells resulted in higher levels of the EGFR protein. Mechanistically, our data reveal that increases in EGFR levels are associated with lower levels of ubiquitination, thus suggesting that in absence of ligand stimulation, EHD3 stabilizes EGFR by decreasing its ubiquitination, dramatically decreases the levels of Cbl constitutively associated, thus suggesting that EHD3 increases the base level of EGFR, by affecting the level of constitutively recruited ubiquitination machinery proteins.

At first examination, the finding that a putative tumor suppressor (*i.e.* EHD3) increases the levels of an oncogene (*i.e.* EGFR) appears counterintuitive. Although their significance is still unclear, similar effects have been reported whereby proteins involved in intracellular trafficking induce the levels of EGFR [35]. In other respects, it is becoming increasingly clear that EGFR can undergo ligand and kinase-independent functions [25, 36, 37], and these functions could be antagonistic to what is observed when the receptor is activated by the ligand. Therefore, it remains to be determined whether and how EHD3 regulates ligand-independent functions of EGFR. Therefore, EHD3 affects EGFR at two seemingly distinct levels: expression level and activation. We have obtained interesting results that are very informative in this regard. First, using protein extracts from U251tetEhd3 cells grown *in vivo* as xenografts and thus exposed to the animal's growth factors, we found that EHD3 expression ultimately results in the inactivation of EGFR, as evidenced by a lower phosphorylation. Second, we observed that EHD3 decreases the constitutive activation of the EGFRvIII mutant, an endocytic trafficking-impaired mutant that escapes signal attenuation and is constitutively active independently from the ligand. The effect of EHD3 doesn't appear to be due to a lower level of expression of EGFRvIII, but rather to a decrease in its activation. While the mechanisms of this regulation await further elucidation, it shows that EHD3 inactivates both wild type EGFR and mutant EGFRvIII.

These findings prompted us to hypothesize that the function of EHD3 might be different in presence of EGFR ligand activation. Indeed, we found that, although EHD3 increases the level of EGFR protein in non-stimulated cells, upon EGF ligand stimulation, the attenuation of EGFR phosphorylation is more dramatic and the total level of EGFR is decreased more rapidly when EHD3 expression is restored than in the EHD3-devoid control cells. This further supports the idea that EHD3 regulates not only EGFR expression but also its signal attenuation, and that it regulates EGFR both ligand-dependently and −independently.

When we assessed how the regulation of EGFR activation and signal attenuation by EHD3 impacts signal transduction, we were surprised to observe that following ligand stimulation, neither AKT nor ERK, two major signaling targets of EGFR activation, was significantly changed. Based on our findings that EHD3 regulates the trafficking of EGFR, we thus questioned whether EHD3 affects any of these signaling pathways in a spatially-regulated manner. We selectively inhibited signaling that originates from the cytoplasmic membrane, to allow specific examination of the endosomal signaling of EGFR [27], and showed that the endosomal activation of EGFR, as well as activation of ERK and AKT was significantly decreased in presence of EHD3. Differential signaling from various subcellular compartments is an important, although not very well studied, process. In particular, it has been shown that EGFR can undergo endosomal signaling [13, 14, 17, 26]. Although EGFR endocytic internalization followed by lysosomal degradation is considered as a process of attenuation of the receptor's signaling, it has now become acknowledged that endosomes are not used only as cargo stations, and that signaling continues from activated EGFR as well as other receptors continues from their location at different endosomal compartments until their degradation [13, 17, 21, 26, 38–40]. The observed differential regulation by EHD3 of EGFR endosomal signaling could explain another interesting finding, *i.e.* that EHD3 enhances growth inhibitory effects of high concentrations of EGF. Although EGF is known for its mitogenic functions, early studies have shown that in many cell lines and contexts it can also induce cell growth inhibition [28–32]. The mechanisms underlying this effect are poorly understood and their deciphering could have an important impact on EGFR-targeted therapy. It appears that these mechanisms involve, at least partially, the receptor frequency and occupancy [41]. Also, differential activation of the ERK MAP kinase pathway is important in the regulation of this dual function of EGF [42–44]. In particular, low and high EGF doses have been reported to have opposite effects on cell growth and gene expression [31, 32, 45] and to be associated with different EGFR auto-phosphorylation [46]. Our data suggest that EHD3 attenuates ERK and more significantly AKT signaling triggered by EGFR activation in the endosomal compartment. Although both pro-apoptotic [47] as well as pro-survival functions [27, 48, 49] have been associated with endosomal signaling, these reports support our findings that EHD3 regulates the cell growth response to EGF by regulating endosomal signaling of EGFR. The spatio-temporal integration of signal transduction is at the mechanistic heart of biological processes in normal homeostasis as well as diseases such as cancer. A major way of achieving such regulation is by modulating receptor trafficking from entry point endocytic internalization to recycling or degradation. The role of intracellular trafficking in cancer is still insufficiently understood.

Endocytic trafficking of EGFR is an essential event in the process of ligand-induced EGFR signal attenuation and degradation. We found that, upon EGF ligand stimulation, the pattern of EGFR trafficking is altered by EHD3 expression, so as to reduce its perinuclear accumulation reminiscent of the recycling compartment. These data suggest that EHD3 attenuates EGFR signaling by regulating its endosomal sorting and ligand-dependent activation. Based on the reported role of EHD proteins in endosomal recycling, we sought to elucidate the trafficking events involved in the effects of EHD3 on the attenuation of EGFR activation. Using two inhibitors, a vesicular trafficking inhibitor Mon A, and the lysosomal degradation inhibitor Chloroquine, we unraveled a strong role for EHD3 in EGF-dependent EGFR degradation; EHD3 plays a major role in the attenuation of EGFR activation via enhancing the lysosomal degradative process. Evidence supports a role for ubiquitination in EGFR lysosomal degradation [50–53]. As a result of EGF ligand stimulation, subsequent EGFR ubiquitination constitutes a sorting signal that favors lysosomal degradation to the expense of the recycling pathway [24, 54–56]. Our data suggests that EHD3 promotes ligand-dependent ubiquitination and lysosomal degradation of EGFR. A mechanistic model is that EHD3 attenuates EGFR activation by accelerating its trafficking out of the endosomal compartment and increasing its lysosomal degradation. This process appears to involve a dramatic acceleration and increase of its ubiquitination. Additional investigations will be required to identify the mechanisms of regulation by EHD3 of the sorting and degradation of EGFR. EHD3 has been shown to be involved in endosomal recycling and retrograde transport [11, 12, 57] and here we provide the first evidence that it is also involved in sorting endosomes to the degradative compartment. Alternatively, there remains the possibility of an indirect effect whereby EHD3 might disturb the endosomal compartments' morphology, thus promoting a different redistribution of endosomal proteins, and therefore causing EGFR to accumulate in late endosomes and lysosomes while reducing its recycling to the cell surface. This effect would be consistent with the reported role of EHD3 in maintaining the Golgi morphology [12].

In summary, our data suggest that EHD3 might undergo its tumor suppressor functions at least in part through promoting the sorting of activated EGFR to the lysosomal degradative compartment and spatio-temporal regulation of EGFR signaling via ERK and AKT. Further elucidation of this process will impact not only the understanding of EGFR signaling and functions, but also our view of the use of EGFR-targeting therapies in gliomas.

## MATERIALS AND METHODS

### Reagents

Tetracycline-free Fetal Bovine Serum was purchased from Clontech. Dulbecco's modified Eagle's medium was purchased from Invitrogen. Rabbit anti-EGFR (EGF Receptor (D38B1) XP^®^ Rabbit mAb #4267) and Rabbit anti-phosphoEGFR (Phospho-EGF Receptor (Tyr1068) (D7A5) XP^®^ Rabbit mAb #3777; Phospho-EGF Receptor (Tyr845) (D63B4) Rabbit mAb) antibodies were from Cell Signaling, Inc. Mouse anti-EGFR (clone 13A9) was a generous gift from Genentech Inc. The anti-EGFRvIII antibody was previously described [58]. For immunoblotting detection of EHD3, we used either a mouse monoclonal antibody (Clone RR-L, Santa Cruz Biotechnologies) or our custom-made rabbit polyclonal antibody [8]. Antibodies against Cbl, phospho-Cbl, Chk, ERK, phospho-ERK, AKT, phospho-AKT, GAPDH and Actin were from Cell Signaling.

### Glioma cell lines

The sublines U251tetEHD3 and U87tetEHD3 cell lines derived from the glioma cell lines U251 and U87MG respectively to express EHD3 upon Doxycycline (Dox) induction were previously described [8]. The cells were authenticated by the WSU Applied Genomics Technology Center (AGTC)(http://agtc.wayne.edu/cell-lines.php) by short tandem repeat profiling using the Promega GenePrint 10 kit. These cells were maintained in DMEM with l-glutamine (Life Technologies, Inc., Invitrogen Corp.), 10% Tet-free fetal bovine serum (Clontech) and 1% penicillin-streptomycin (Life Technologies, Invitrogen) and grown at 37°C. Induction of EHD3 expression is obtained by treating cells with 2 mg/ml of Dox for the indicated time. U251MG cells conditionally expressing the EGFRvIII mutant using the T-Rex Tet-on system (Invitrogen) were a kind gift from Dr Amyn Habib (University of Texas Southwestern Medical Center) and were described previously [59].

### Real time RT-PCR

Total RNA was isolated from glioma cell lines using the RNAquous-4 PCR Kit (Ambion) according to the manufacturer's instructions. For real time quantitative RT-PCR, we followed the SYBR green protocol, using the iTaq Fast SYBR Green Supermix with ROX (Biorad) as directed by the manufacturer. *Egfr* and control *Tbp* amplification was done using primers from realtimeprimers.com (*VHPS-10346 and* VHPS-9111 *respectively)*. All experiments were carried out at least in triplicates.

### Cell lysate preparation and immunoblotting

Cells were washed twice with phosphate buffered saline (PBS) and scraped in ice-cold lysis buffer [20 mM Hepes, pH 7.4, 1% NP40, 2 mM EDTA, 100 mM NaF, 10 mM Pyrophosphate, 1 mM sodium vanadate] containing 1× protease inhibitor cocktail. Then, the cell lysates were solubilized by sonication and cleared by centrifugation at 14,000 rpm for 10 min at 4C.

### MTT assay

The 3-(4,5-dimethylthiazo-2-yl)-2,5-diphenyltetrazolium (MTT) metabolic assay was used to assess cell growth. Briefly, exponentially growing cells were seeded in 96-well micro-plates for 24 h. For the inducible system, cells were induced by Doxycycline at 1 mg/ml. Survival was evaluated every day by replacing the culture media with 50 ml of 2.5 mg/ml MTT (sigma, St Louis, MO), in PBS pH7.5. After 1 h of incubation at 37C in the dark, MTT was replaced with 100 ml of solubilization solution (10% triton X100, and 1 N HCl in anhydrous isopropanol). Absorbance was determined at 570 nm with a microplate reader (Biorad). The numbers indicated are the mean result of three independent experiments (mean ± SD).

### Immunofluorescence microscopy

Cells were cultured on glass cover slips overnight. They were incubated for 10 min on ice with the clone 13A9 anti-EGFR antibody (Genentech Inc.) before stimulation with EGF (50 ng/mL).

At different time points, cells were washed with cold PBS, fixed with 4% formaldehyde in PBS for 15 minutes at room temperature, PBS-washed, permeabilized with 0.2% Triton-X in PBS, washed again with PBS. Incubation was performed using a blocking buffer [2% BSA, 2% Normal Goat Serum and 02% Gelatin in PBS] for 30–60 min at room temperature. Cells were then incubated with a fluorescently labeled secondary antibody (Alexafluor-594-conjugated, Molecular Probes) in blocking buffer for 60 min at room temperature. After a wash with 01% BSA in PBS, 4 times 5 min, and an additional 2 times 5 min in PBS, excess liquid was carefully removed. Coverslips were mounted onto slides using the Mowiol mounting reagent (Polysciences Inc.) as per the manufacturer's instructions. Slides were stored at 4°C and examined the next day using a Leica TCS SP5 Laser (Point) Scanning Confocal microscope. Confocal microscopy was done at the Microscopy, Imaging and Cytometry Resources Core at Wayne State University, School of Medicine.

### Statistical analysis

Statistics were conducted using GraphPad Prism 5.0 for Windows (GraphPad Software) and tests were done with a Student *t* test (*P < 0.05, **P < 0.01, ***P < 0.001). *P* values less than 0.05 were considered to be statistically significant. Results are displayed as averages with error bars indicating standard deviations (SD). Signal intensities were quantified using the Image Studio Lite software (LI- COR Biosciences).

## SUPPLEMENTARY MATERIALS


