# Supplementary material for: Spatio-temporal regulation of EGFR signaling by the Eps15 homology domain-containing protein 3 (EHD3)
Source: Oncotarget. 2016 Nov 1;7(48):79203–16. doi: 10.18632/oncotarget.13008 (PMC5346708; doi:10.18632/oncotarget.13008)
Supplement: Supplementary file 1 [file oncotarget-07-79203-s001.pdf]

Spatio-temporal regulation of EGFR signaling by the Eps15 homology domain-containing protein 3 (EHD3)

Supplementary Materials

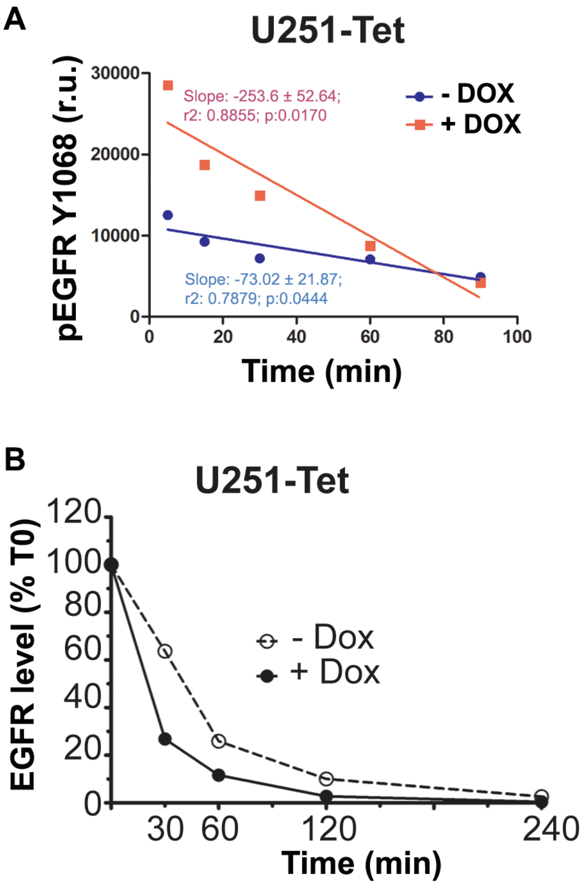

Supplementary Figure S1: Effect of EHD3 on ligand-dependent activation of EGFR. (**A**) Linear regression of the levels of pEGFR Y1068 at 5–90 min, generated by GraphPad Prizm 5.0, using densitometric quantitation data from Figure 3A. (**B**) Densitometric quantitation of a representative immunoblot of EGF ligand-stimulated U251tetEhd3 cells at different time points. The results are shown as relative units (r.u.) of the EGFR levels in Dox-induced versus the control without Dox treatment, the time 0 (T0) level representing a 100% value.

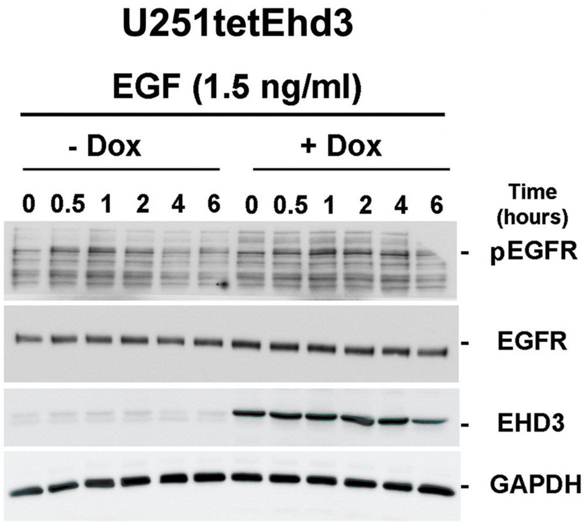

Supplementary Figure S2: Effect of low dose EGF stimulation on EGFR activation in U251tetEhd3 cells. Cells were induced (+Dox) or not (–Dox) to express EHD3 by Dox treatment, serum starvation-primed overnight prior to stimulation with EGF (1.5 ng/ml) at different time points. Times are more extended than used in high dose EGF stimulation. Whole cell lysates were analyzed by immunoblotting for the levels of total EGFR, tyrosine phosphorylated EGFR (pEGFR), EHD3 and GAPDH.

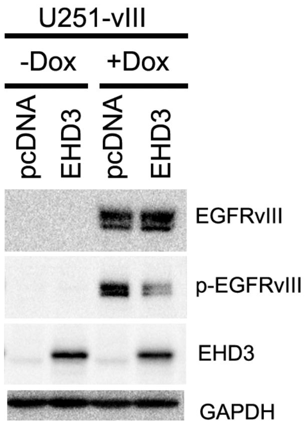

Supplementary Figure S3: Effect of EHD3 on constitutive activation of EGFRvIII mutant. A clone of U251MG cells was induced to express EGFRvIII (molecular weight 145 kDa) by Dox treatment (Dox, 2 µg/ml) and concomitantly transfected with a control plasmid (pcDNA) or a plasmid encoding EHD3 (EHD3). Whole cell lysates were analyzed by immunoblotting for the levels of total EGFRvIII, tyrosine phosphorylated EGFRvIII (pEGFRvIII), EHD3 and GAPDH.

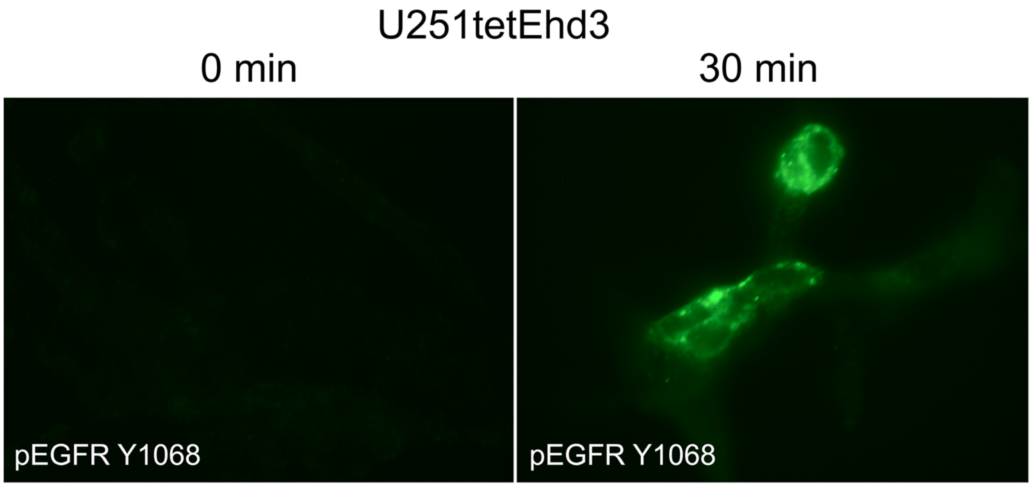

Supplementary Figure S4: Selective activation of endosomal EGFR. U251tetEhd3 cells were serum starved for 24 h, pre-treated with the EGFR tyrosine kinase inhibitor AG1478, prior to treatment on ice with EGF, thus allowing the endosomal internalization of non-activated EGF-EGFR complexes without triggering membrane-originated signaling. The endosomal EGFR signaling was subsequently activated by removing AG-1478 from the medium and incubating at 37C. Subsequently, immunofluorescent labeling of the phosphorylated EGFR was performed using an anti-phospho-EGFR Y1068 antibody (Cell Signaling Inc.) and an AlexaFluor-488-conjugated secondary antibody (Molecular Probes).
